# Common Variation in *ISL1* Confers Genetic Susceptibility for Human Congenital Heart Disease

**DOI:** 10.1371/journal.pone.0010855

**Published:** 2010-05-26

**Authors:** Kristen N. Stevens, Hakon Hakonarson, Cecilia E. Kim, Pieter A. Doevendans, Bobby P. C. Koeleman, Seema Mital, Jennifer Raue, Joseph T. Glessner, John G. Coles, Victor Moreno, Anne Granger, Stephen B. Gruber, Peter J. Gruber

**Affiliations:** 1 Department of Epidemiology, University of Michigan, Ann Arbor, Michigan, United States of America; 2 The Children's Hospital of Philadelphia, Philadelphia, Pennsylvania, United States of America; 3 Department of Cardiology, UMC Utrecht, Utrecht, The Netherlands; 4 Department of Medical Genetics, UMC Utrecht, Utrecht, The Netherlands; 5 Heart Center, Hospital for Sick Children, Toronto, Ontario, Canada; 6 Biostatistics and Bioinformatics Unit, IDIBELL-Catalan Institute of Oncology and University of Barcelona, L'Hospitalet del Llobregat, Barcelona, Spain; 7 Department of Internal Medicine, University of Michigan, Ann Arbor, Michigan, United States of America; 8 Department of Human Genetics, University of Michigan, Ann Arbor, Michigan, United States of America; 9 Penn Cardiovascular Institute, University of Pennsylvania School of Medicine, Philadelphia, Pennsylvania, United States of America; 10 Institute for Regenerative Medicine, University of Pennsylvania School of Medicine, Philadelphia, Pennsylvania, United States of America; Ohio State University Medical Center, United States of America

## Abstract

Congenital heart disease (CHD) is the most common birth abnormality and the etiology is unknown in the overwhelming majority of cases. ISLET1 (ISL1) is a transcription factor that marks cardiac progenitor cells and generates diverse multipotent cardiovascular cell lineages. The fundamental role of ISL1 in cardiac morphogenesis makes this an exceptional candidate gene to consider as a cause of complex congenital heart disease. We evaluated whether genetic variation in *ISL1* fits the common variant–common disease hypothesis. A 2-stage case-control study examined 27 polymorphisms mapping to the *ISL1* locus in 300 patients with complex congenital heart disease and 2,201 healthy pediatric controls. Eight genic and flanking *ISL1* SNPs were significantly associated with complex congenital heart disease. A replication study analyzed these candidate SNPs in 1,044 new cases and 3,934 independent controls and confirmed that genetic variation in *ISL1* is associated with risk of non-syndromic congenital heart disease. Our results demonstrate that two different *ISL1* haplotypes contribute to risk of CHD in white and black/African American populations.

## Introduction

CHD affects 1 in 20 live births, 1 in 100 of which require an intervention [1], [2]. Recognizable chromosomal variants account for 13% of all CHD patients although high-resolution technologies may uncover more subtle defects [3]. Additionally, mutations in a few genes, such as *GATA4* and *NKX2–5*, have been associated with rare-monogenic disorders that include a cardiac phenotype in humans [4], [5], [6]. However, no common genetic variants have been robustly associated with the risk of non-syndromic, complex CHD. Here we describe the first such association.

During vertebrate cardiac development, the 3-dimensional structure of the heart is formed from the differentiation and interaction of multiple tissue derivatives, or fields [7]. The primary and secondary heart fields of the embryonic disc give rise to the intracardiac structures of the heart under the influence of adjacent tissues [8]. The secondary heart field provides an especially important source of cells directing complex morphogenesis and is the source of cardiac progenitor cells marked by the transcription factor ISL1. These can be isolated from postnatal hearts of rodents and humans that maintain the ability to differentiate into multiple cardiac lineages [9], [10]. Although mice deficient in *Isl1* harbor defects in cardiac morphogenesis, the role of *ISL1* in human congenital heart disease is unknown [11], [12].

## Results

We conducted a two-stage candidate gene study to test the hypothesis that germline common genetic variants in *ISL1* confer susceptibility to non-syndromic human CHD. The stage 1 study was comprised of 300 CHD cases (white n = 160, black/African American n = 70, other/unknown = 70) and 2,201 CHD-free controls (white n = 2091, black/African American n = 110). Cases were children diagnosed with complex, non-syndromic CHD, all of which required operative repair. Guided by lineage-tracing analyses in rodents, cases were defined by diseases representative of secondary heart field defect phenotypes [11], [13], [14]. Defects of the second heart field are potentially pathogenic in anatomic defects of both the right and left sides of the normal heart due the contribution of secondary heart field derivatives in both inflow and outflow tracts. These include defects of atrial septation, ventricular septation, conus positioning, and great vessel alignment (Fig. S1).

**Figure 1:**
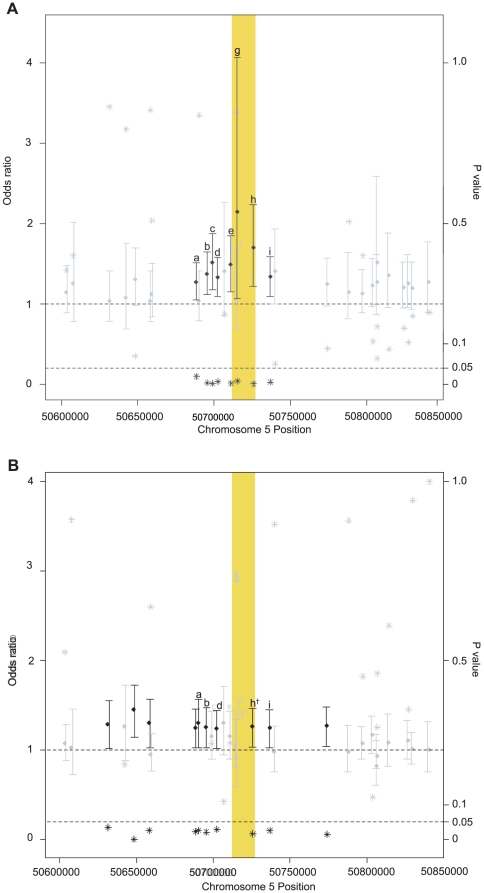
*ISL1* SNP associations with CHD on chromosome 5. a) Analysis of SNP data within and surrounding *ISL1* in stage 1 yielded 8 SNPs that were significantly associated with CHD in an ethnically heterogeneous US population. ORs, 95%CIs and P values significant at = 0.05 are depicted in black. Non-significant ORs, 95% CIs and P values are depicted in grey. The yellow highlighted region indicates the location of ISL1 on chromosome 5. Labeled SNPs: (a) rs6867206, (b) rs4865656, (c) rs6869844, (d) rs2115322, (e) rs6449600, (f) rs3762977, (g) IVS1+17C>T, (h) rs1017, (i) rs6449612. b) Analysis of SNP data within and surrounding *ISL1* in stage 2 US whites yielded10 SNPs that were significantly associated with CHD in an initial analysis of an ethnically heterogeneous US population. ORs, 95%CIs and P values significant at = 0.05 are depicted in black. Non-significant ORs, 95% CIs and P values are depicted in grey. The yellow highlighted region indicates the location of ISL1 on chromosome 5. Labeled SNPs: a) rs6867206, b) rs4865656, c) rs6869844, d) rs2115322, e) rs6449600, f) rs3762977 †, g) IVS1+17C>T †, h) rs1017 †, i) rs6449612. † SNP genotypes determined by imputation.

We analyzed 30 SNPs spanning a 237 kb region around *ISL1* on chromosome 5q11.1, selected to capture variation in this region based upon linkage disequilibrium patterns in subjects of European ancestry (http://www.hapmap.org). No genome-wide data were available for this hypothesis-driven, candidate gene study. Eight individual SNPs (rs6867206, rs4865656, rs6869844, rs2115322, rs6449600, IVS1+17C>T, rs1017, rs6449612) were significantly associated with risk of CHD at the α = 0.05 level (Fig. 1A) located within a single LD block. Indeed, HapMap data demonstrate D' = 1 between three of these SNPs (rs6869844, rs6449600, rs6449612) and each of the four Hapmap published SNPs within *ISL1* (rs3792733, rs2288468, rs3811911, rs991216). The moderate magnitudes of association seen at these SNPs (OR = 1.32–2.30) were consistent with those expected under the common disease-common variant hypothesis. Furthermore, the closest gene to *ISL1* is located in a different LD block more than 540 kb upstream (*PARP8*), reducing the likelihood that these SNPs are capturing an association between a gene other than *ISL1* and risk of CHD. Of the six *ISL1*-flanking SNPs, rs6869844 remained statistically significant after adjustment for multiple testing (*P* = 0.039 with Bonferroni correction for 30 SNPs). Located 15.7 kb 5′ of *ISL1*, rs6869844 was associated with a 50% increase in risk for each additional T allele in a log-additive model (Odds ratio (OR) = 1.51, 95% confidence interval (CI) = 1.18–1.95).

Three SNPs analyzed in stage 1 (rs3762977, IVS1+17C>T, rs1017) were located within the *ISL1* gene in the 5′UTR, intron 1, and the 3′ UTR, respectively (Fig. S2). To diminish the potential for confounding population stratification, we first restricted our stage 1 analyses to white cases with non-syndromic CHD and white controls (n = 100 cases, 576 controls) with genotype data available for these SNPs. IVS1+17C>T was associated with a more than two-fold increase in risk among whites with the C/T genotype (OR = 2.30, 95% CI 1.12–4.70, *P* = 0.023) (Table 1). Rs1017 was highly significant in a log additive model, with an 81% increase in risk associated with each additional copy of the T allele (OR = 1.81, 95% CI 1.29–2.54, *P* = 0.0007). Dominant and recessive models for rs1017 were also highly significant. Children with the A/T or T/T genotype had a 2.28-fold increase in risk compared to children with the A/A genotype (OR = 2.28, 95% CI 1.35–3.87, *P* = 0.002). Similarly, children with the T/T genotype had a 2.21-fold increase in risk compared to children with the A/A or A/T genotype (OR = 2.11, 95% CI 1.17–3.80, *P* = 0.013).

**Table 1:** *ISL1* SNP associations with risk of congenital heart disease in US whites.

Genotypes	Controls [n (%)]	Cases [n (%)]	OR [95% CI]	*P* value
**Stage 1**				
*rs3762977*				
A/A	329 (75.3)	65 (79.3)	1.00	
A/G	102 (23.3)	15 (18.3)	0.74 (0.41/1.36)	0.338
G/G	6 (1.4)	2 (2.4)	1.68 (0.33/8.55)	0.527
		log-additive:	0.87 (0.52/1.47)	0.607
*IVS1+17C>T*				
C/C	402 (93.1)	70 (85.4)	1.00	
C/T	30 (6.9)	12 (14.6)	2.30 (1.12/4.70)	0.023
*rs1017*				
A/A	182 (42.8)	21 (25.3)	1.00	
A/T	192 (45.2)	43 (51.8)	1.94 (1.11/3.40)	0.020
T/T	51 (12.0)	19 (22.9)	3.23 (1.61/6.46)	0.0009
		log-additive:	1.81 (1.29/2.54)	0.0007
**Stage 2 validation**				
*rs3762977*				
A/A	1128 (18.1)	202 (76.2)	1.00	
A/G	289 (20.0)	58 (21.9)	1.12 (0.82/1.54)	0.481
G/G	28 (1.9)	5 (1.9)	1.00 (0.38/2.62)	0.997
		log-additive:	1.08 (0.82/1.42)	0.571
*IVS1+17C>T*				
C/C	1334 (92.3)	246 (92.8)	1.00	
C/T	111 (7.0)	19 (7.2)	0.93 (0.56/1.54)	0.773
A/A	591 (40.9)	91 (34.3)	1.00	
A/T	672 (46.5)	128 (48.3)	1.24 (0.93/1.66)	0.148
T/T	182 (12.6)	46 (17.3)	1.64 (1.12/2.43)	0.013
		log-additive:	1.27 (1.05/1.54)	0.013

We then delineated the patterns of risk in these populations by using the expectation maximization (EM) method to estimate haplotypes and risk of CHD from the 6 *ISL1*-flanking SNPs (rs6867206, rs4865656, rs6869844, rs2115322, rs6449600, rs6449612) and the 3 SNPs within *ISL1* (rs3762977, IVS1+17C>T, rs1017). The three SNPs within *ISL1* most effectively captured risk of CHD. In stage 1 whites, an additive model fit the data well (global haplotype association *P* = 0.0008). Two haplotypes, A-C-T and A-T-T (rs3762977- IVS1+17C>T -rs1017), were strongly associated with CHD risk. A child's risk of CHD was 2.01 times greater with each copy of the A-C-T haplotype compared to the A-C-A haplotype (95%CI 1.35–3.00, *P* = 0.0006) and 3.29 times greater with each copy of the A-T-T haplotype (95% CI 1.51–7.16, *P* = 0.0027).

To independently validate our findings, we studied *ISL1* variation in a second, independent analysis of populations from the US, Canada, and the Netherlands. Stage 2 cases and controls were completely distinct from those in initial stage 1. The stage 2 white population consisted of 995 cases (US n = 265, Canada n = 94, Netherlands n = 636) and 2089 controls (North America n = 1446, Netherlands n = 643). Single SNP analyses in the stage 2 US white population confirmed the association at 10 SNPs within and around *ISL1* (Fig. 1B). Specifically, of the 3 SNPs within *ISL1*, rs1017 was significantly associated with risk of CHD in a log-additive model (Table 1). Each copy of the T allele at rs1017 increased a child's risk of CHD by 27% (OR = 1.27, 95% CI 1.05–1.54, *P* = 0.013).

**Figure 2:**
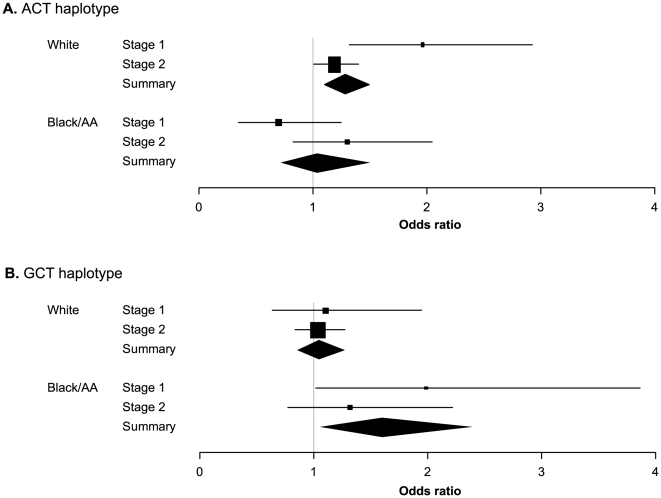
*ISL1* haplotypes and risk of congenital heart disease by race/ethnicity. a) The A-C-T risk haplotype in white stage 1 (US) and stage 2 (US, Canada, Netherlands) populations. Odds ratios (95% CIs) for each stage are denoted by black boxes (gray lines). Summary OR estimates are represented by black diamonds, where diamond width corresponds to 95% CI bounds. Box and diamond heights are inversely proportional to precision of the OR estimate. b) The G-C-T risk haplotype in black/African American stage 1 (US) and stage 2 (US) populations. Odds ratios (95% CIs) are denoted as in 2a.

Haplotype analysis in the stage 2 white population confirmed that the A-C-T haplotype is significantly associated with risk among whites (Global *P* = 0.00003). Each copy of the A-C-T haplotype conferred an 18% increase in risk (OR = 1.18, 95% CI 1.00–1.39, *P* = 0.0485) compared to the A-C-A haplotype. Combined analysis of both stage 1 and stage 2 for the A-C-T haplotype in whites was highly significant (Tables 2, S4, S5; Fig. 2), with a 27% increase in risk of CHD (95% CI 1.09–1.48, *P* = 0.0018).

**Table 2:** Summary *ISL1* haplotype association with risk of CHD in all white populations (stage 1 & stage 2).

Haplotypes	rs3762977	IVS1+17C>T	rs1017	Frequency (%)	OR [95% CI]	*P* value
1	A	C	A	0.645	1.00	
2	A	C	T	0.192	1.27 (1.09/1.48)	0.0018
3	G	C	T	0.098	1.07 (0.88/1.30)	0.5068
4	A	T	T	0.034	1.04 (0.75/1.44)	0.8216
5	G	C	A	0.022	1.10 (0.78/1.53)	0.5928
	Global haplotype association					0.000004

Rare estimated haplotypes (cumulative frequency = 0.0099) not shown.

The precise distribution of CHD diagnoses was different between stage 1 and stage 2 populations (Fig. S1). However, hypoplastic left heart syndrome (HLHS) and D-transposition of the great arteries (D-TGA) were the most common diagnoses in both stages. To ensure that the stage 2 validation of our original findings was not influenced by the differences among case populations, we performed a subset analysis to include only the most frequent diagnoses in both stages, HLHS and D-TGA. Associations with rs1017 and the A-C-T haplotype in stage 1, stage 2, and combined analyses were consistent with analyses utilizing cases of all secondary heart field defects in both magnitude and significance of association (Tables S4, S5, and S6). This indicates that risk of CHD is consistently associated with common genetic variation in *ISL1* in white populations whether considering all secondary heart field defects or subsets of these diagnoses.

To understand the role of *ISL1* variation in other populations, we investigated these 3 SNPs in the stage 1 black/African American cases and controls, using the exact same phenotypic definitions for cases with non-syndromic CHD (n = 54 cases, 110 controls). Compared to whites, analysis at these three loci demonstrated a different pattern of association between *ISL1* and risk of CHD. While no association was observed at rs3762977 in whites, black/African American children were at a more than 2-fold increase in risk for each additional copy of the G allele at this locus (OR = 2.21, 95%CI 1.15–4.24; *P* = 0.017). Variation at IVS1+17C>T was extremely rare among blacks/African Americans with only 1 heterozygous control and 0 heterozygous cases, and no association between rs1017 and risk of CHD was observed (OR = 1.08, 95%CI 0.66–1.76; *P* = 0.756). However, as with the single SNP analyses, haplotype analysis showed that the black/African American population demonstrated a distinct pattern of risk at the *ISL1* locus. The A-C-T haplotype was not associated with increased risk of CHD among blacks, and the A-T-T haplotype was not identified in any cases or controls of black/African American ancestry. In contrast, the G-C-T haplotype was associated with a 2-fold increase in risk of CHD (OR = 1.99, 95%CI 1.02–3.87; *P* = 0.043).

To independently validate our findings in blacks/African Americans, we analyzed a distinct set of 49 US black/African American cases and 1,845 US black/African American controls. In this stage 2 population, the relative risk for rs3762977 was consistent with that seen in stage 1 blacks/African Americans (OR = 1.20, 95% CI 0.74–1.95, *P* = 0.457), although not statistically significant. Similarly, the G-C-T haplotype did not reach statistical significance among blacks/African Americans in stage 2, but the relative risk for this haplotype was consistent with that seen in stage 1 (OR = 1.28, 95% CI 0.75–2.19, *P* = 0.359). Importantly, the G-C-T haplotype was significantly associated with risk of CHD in a summary analysis of stage 1 and stage 2 blacks/African Americans, where each copy of this haplotype conferred a 57% increase in risk (95% CI 1.07–2.30, *P* = 0.0216) (Fig. 2).

## Discussion

Our results demonstrate that two different *ISL1* haplotypes contribute to risk of CHD in white and black/African American populations. These data provide strong evidence that congenital heart disease is consistent with the common variant–common disease hypothesis in two ethnically distinct populations. Further work is necessary to determine whether these two haplotypes capture ancestrally distinct causative mutations or are in linkage disequilibrium with a single disease-causing mutation. Our observations of different risk haplotypes in white and black/African American populations is intriguing and suggests that different risk alleles are present in the ISL1 locus within these populations. This provides an opportunity for identifying causal variants through subsequent studies with admixture mapping or deep sequencing within these two patient populations.

The biologic rationale is compelling: ISL1 is a transcription factor that marks cardiac progenitor cells and controls secondary heart field differentiation, and new evidence suggests that purified populations of ISL1+ progenitor cells are capable of self-renewal and expansion into cardiomyocytes, smooth muscle, and endothelial lineages [15]. In addition to providing new insight into the variety of congenital heart disease phenotypes that can be produced from second heart field defects in humans, our observations also may provide the basis for a more integrated understanding of the molecular basis of human congenital heart disease.

## Materials and Methods

### Sample collection

United States cases and controls were recruited from the Children's Hospital of Philadelphia (CHOP) between 12/12/2003 and 08/25/2008 on a protocol approved by the Institutional Review Boards of CHOP and the University of Michigan, and parents provided written informed consent. 31.6% (613/1939) of all eligible cases seen at the CHOP cardiac center in this time period participated in this study. Cases and controls from Toronto and the Netherlands were recruited on institution-specific protocols, and were also approved by the IRBs of CHOP and the University of Michigan. Cases were children with complex congenital heart disease requiring surgical repair. Controls were patients without congenital heart disease recruited through either the CHOP Health Care Network by CHOP clinicians and nursing staff or through UMC Utrecht. The controls were screened by counselors who evaluated by patient history for a absence of structural heart disease. All patients had been evaluated by a medical doctor. Ethnicity for cases was determined by self-report and principal components analysis, and ethnicity for controls was determined by principal components analysis. This analysis was performed for all stage 2 subjects of unknown ethnicity as well as a representative subset of stage 1 cases and controls of known ethnicity (Fig. S3). The first two principal components were plotted for stage 1 cases of known ethnicity, demonstrating that the first principal component distinguished between white and blacks/African American cases using a cutoff of PC1≤0.025 to define ethnicity as white and PC1>0.025 to define ethnicity as black/African American. Similarly, the first principal component sufficiently distinguished between stage 1 white and blacks/African American controls, using a cutoff of PC1≤0.0059 to define ethnicity as white and PC1>0.0059 to define ethnicity as black/African American.

### *ISL1* genotyping

Stage 1 and stage 2 genotypes were requested for 27 *ISL1*-flanking SNPs from the Center for Applied Genomics at CHOP that had been performed using the Illumina HumanHap 550 SNP array. Genotypes for only these 27 SNPs were obtained from the Center for Applied Genomics, and no additional genotypes on this platform were obtained or analyzed. Data was available for these 27 SNPs for US cases and US controls in both stage 1 and stage 2.

Genotypes for the 3 SNPs within *ISL1* (rs3762977, IVS1+17C>T, rs1017) were determined using one of two methods: 1) bidirectional sequencing of *ISL1* exons 1 and 6 or 2) genotype imputation. Stage 1 genotyping of these 3 SNPs was performed using bidirectional Sanger sequencing, accomplished at the University of Michigan sequencing core (Table S1 and Table S2). Stage 2 Canadian and Dutch cases were also genotyped using bidirectional sequencing. Imputation was performed for the 3 SNPs within *ISL1* for US cases and all controls using 97 SNPs surrounding these 3 SNPs. Haplotypes were reconstructed for all 100 SNPs in 484 controls using FastPHASE^14^. These phased, reconstructed haplotypes were then used as the reference haplotypes for genotype imputation using the MACH program [16], [17]. Each genotype at each SNP was associated with a QC score, interpreted as the posterior probability that the imputed genotype represents the true genotype.

Genotyping accuracy by sequencing was assessed with repeat sequencing of a subset of genotypes. Two measures were used to assess imputation error for the three *ISL1* SNPs. The first measure, ε_j_, captures genotyping error, discrepancies with the reference panel, and recurrent mutation [16], [17]. Slightly lower data quality is observed for larger estimates of ε_j_. Values of ε_j_ were small for each of the three SNPs: rs3762977 ε_j_ = 0.0229, IVS1+17C>T ε_j_ = 0.0007, rs1017 ε_j_ = 0.0434. The second measure of imputation error was the agreement between genotypes determined by sequencing and genotypes determined by imputation for a subset of stage 1 cases and controls for whom both genotypes were available. Agreement was measured using the Kappa statistic in SAS (version 9.1) at various QC cutoffs. No QC values for any of the 3 imputed SNPs were less than 0.5. Inclusion of all imputed genotypes (regardless of QC value) resulted in Kappa statistics of at least 0.889 for each of the 3 SNPs, confirming that the genotype imputation method is robust. All genotype frequencies were assessed for departure from Hardy-Weinberg equilibrium in controls.

### Statistical methods

Single SNP analyses were conducted using unconditional logistic regression to calculate odds ratios as implemented in SAS (version 9.1). Haplotypes were estimated and tested for association with CHD using the Haplo.stats package in R (http://cran.r-project.org). Significance testing was adjusted for multiple comparisons with Bonferroni correction, and 95% confidence intervals were calculated from the parameters estimated in logistic regression. Population attributable fractions were calculated using haplotype-specific odds ratios and haplotype frequencies.

### Additional methods for the adjustment of population stratification

We performed additional analyses to determine the sensitivity of our previously described method of defining ethnicity by principal components analysis in stage 2. The alternative classification method we employed was the ANCESTRYMAP [18] program, which uses genotype information from two ancestral populations to estimate admixture in a test population. We used 26 of 136 ancestral informative markers (AIMs) on chromosome 5 for which genotype information for stage 2 subjects was available (Table S3) [19]. None of these 26 SNPs were in linkage disequilibrium with the *ISL1* locus based upon LD patterns in subjects of European ancestry (http://www.hapmap.org). The 26 AIM genotypes for the two ancestral populations, the Centre d'Etude du Polymorphisme Humain (European) and Yoruban (African) HapMap samples, were downloaded from http://www.hapmap.org. We ran the ANCESTRYMAP program [18] using default parameters to obtain estimates of the percent European ancestry for all US stage 2 subjects of unknown ancestry as well as a subset of US stage 1 subjects of known ancestry (Fig. S4). A bimodal distribution of the percent European ancestry was observed among all subjects, which was highly correlated with self-reported ethnicity among stage 1 subjects. Subjects with greater than 65% European ancestry were defined as white, and subjects with less then 65% European ancestry were defined as black/African American. Single SNP and haplotype analyses were performed using ANCESTRYMAP-defined ethnicity, and results were qualitatively similar to those described above.

## Supporting Information

Figure S1 Diagnosis distribution in stage 1 and stage 2 case-control studies. Cases were chosen a priori to represent a wide variety of developmental phenotypes that include developmental structures aberrantly formed as derivatives of the secondary heart field. These diagnostic choices were informed from lineage tracing analyses of Isl1+ progenitor cells in rodents. ALCAPA- anomalous left coronary artery from the pulmonary artery, ASD- atrial septal defect, AVSD- atrioventricular septal defect/AV canal, CCTGA- congenitally corrected transposition of the great arteries, L-TGA- L-transposition of the great arteries, D-TGA- D- transposition of the great arteries, DORV- double outlet right ventricle, HLHS- hypoplastic left heart syndrome, AS-aortic stenosis, AA- aortic atresia, MS- mitral stenosis, MA- mitral atresia, DILV- double inlet left ventricle, TAPVC- total anomalous pulmonary venous connection, TOF- tetralogy of Fallot, VSD- ventricular septal defect.
(0.50 MB EPS)

Figure S2 Chromosome 5 variation in the ISL1 region. The location of ISL1 on chromosome 5 (Build 36) is depicted, where exons of the ISL1 gene are depicted as shaded boxes, the 5′ UTR and 3′ UTR are depicted as white boxes, and introns are represented as black lines. The three SNPs within ISL1 studied in stage 1 and stage 2 are depicted with respect to their location in the gene. The six SNPs flanking ISL1 identified as significantly associated with risk of CHD in stage 1 are indicated along chromosome 5.
(0.42 MB EPS)

Figure S3 Ethnic distribution of cases and controls by cluster analysis. The first two principal components from a principal components analysis utilizing all SNPs on chromosome 5 that are contained within the Illumina HumanHap550 array are plotted for a) stage 1 cases of known ethnicity, where PC1≤0.025 captures white cases and PC1>0.025 captures black/African American cases; b) stage 2 cases of unknown ethnicity, where PC1≤0.025 defines white cases and PC1>0.025 defines black/African American cases; c) stage 1 controls of known ethnicity, where PC1≤0.0059 captures white controls and PC1>0.0059 captures black/African American controls; b) stage 2 cases of unknown ethnicity, where PC1≤0.0059 defines white controls and PC1>0.0059 defines black/African American controls.
(2.84 MB EPS)

Figure S4 ANCESTRYMAP admixture estimation using 26 Ancestral Informative Markers. The distribution of estimated percent European ancestry for a) all stage 1 US subjects of known ethnicity (n = 650), b) stage 1 US whites (n = 251), c) stage 2 US blacks/African Americans (n = 399), and d) stage 2 US subjects of unknown ethnicity (n = 3610). 65% cutoff is represented by a red line. Individuals above 65% European ancestry were defined as white in stage 2 US subjects and below 65% were defined as black/African American.
(0.53 MB EPS)

Table S1 PCR primers & conditions for ISL1 sequencing.
(0.05 MB DOC)

Table S2 Stage 1: ISL1 variation identified by sequencing.
(0.08 MB DOC)

Table S3 Ancestral informative markers available for stage 2 subjects.
(0.05 MB DOC)

Table S4 ISL1 associations with risk of HLHS and TGA in white populations.
(0.03 MB DOC)

Table S5 CHD diagnoses among whites with ACT haplotype.
(0.07 MB DOC)

Table S6 CHD diagnoses among blacks/African Americans with GCT haplotype.
(0.05 MB DOC)
