# Supplementary material for: Common Variation in ISL1 Confers Genetic Susceptibility for Human Congenital Heart Disease
Source: PLoS One. 2010 May 26;5(5):e10855. doi: 10.1371/journal.pone.0010855 (PMC2877111; doi:10.1371/journal.pone.0010855)
Supplement: Table S1 — PCR primers & conditions for ISL1 sequencing. (0.05 MB DOC) [file pone.0010855.s005.doc]

**Table S1. PCR primers & conditions for *ISL1* sequencing**

| **Exon** | **Primer Sequence** | **T_a_°*** |
| --- | --- | --- |
| 1 | F: 5' GAG CAG CGC CAC AGG AGG C 3' | 62 |
|  | R: 5' CTT GGC ACC TCA GCC TGT GC 3' |  |
| 2 | F: 5' GTA GGA AGT AAA CGG TTA GTC 3' | 56 |
|  | R: 5' CTT GTA TGA CTA CAC TGA GGC 3' |  |
| 3 | F: 5' AGT GCC GGC CTG AAG TGA C 3' | 62 |
|  | R: 5' ACA GGC TGG CTT AAC CTG G 3' |  |
| 4 | F: 5' AAG CGA GCC TCC AGC CCA G 3' | 62 |
|  | R: 5' GTG CGA TCC TGC GTA CCA G 3' |  |
| 5 | F: 5' AAC ATG TTG GGA TTG GTT GGG 3' | 56 |
|  | R: 5' TTC CAT CTG GGA GCT GAC AC 3' |  |
| 6 | F: 5' ATG AAT ACT ATT CCA GTG TCC 3' | 56 |
|  | R: 5' GTT TGG CAA GGC AAT GAC C 3' |  |
|  | F: 5' TCT AGT CCA TCC TAA TCT G 3' | 56 |
|  | R: 5’ AAA GTG GCA AGT CTT CCG AC 3’ |  |

* Cycling conditions for all primer sets: Initial denaturation at 95°C for 10min, 30 cycles of 95°C for 30sec, T_a_°C for 30sec, 72°C for 1min, final extension at 72°C for 10min.
