# Supplementary material for: Common Variation in ISL1 Confers Genetic Susceptibility for Human Congenital Heart Disease
Source: PLoS One. 2010 May 26;5(5):e10855. doi: 10.1371/journal.pone.0010855 (PMC2877111; doi:10.1371/journal.pone.0010855)
Supplement: Table S2 — Stage 1: ISL1 variation identified by sequencing. (0.08 MB DOC) [file pone.0010855.s006.doc]

**Table S2. Stage 1: *ISL1* variation identified by sequencing**

|  | **Polymorphism** | **Controls** | | | **Cases** | | |
| --- | --- | --- | --- | --- | --- | --- | --- |
| Exon 1 | rs3762977 | AA | AG | GG | AA | AG | GG |
|  |  | 329 | 102 | 6 | 136 | 39 | 4 |
| Exon 1 | EX1+67G>C | GG | GC | CC | GG | GC | CC |
|  |  | 432 | 4 | 0 | 176 | 1 | 0 |
| Exon 1 | EX1+192C>G | CC | CG | GG | CC | CG | GG |
|  |  | 438 | 0 | 0 | 179 | 1 | 0 |
| Exon 1 | rs36216897 | AA | AG | GG | AA | AG | GG |
|  |  | 418 | 15 | 0 | 175 | 3 | 0 |
| Exon 1 | EX1-269G>A | GG | GA | AA | GG | GA | AA |
|  |  | 424 | 9 | 0 | 178 | 2 | 0 |
| Exon 1 | EX1-215T>G | TT | TG | GG | TT | TG | GG |
|  |  | 432 | 0 | 0 | 178 | 1 | 0 |
| Exon 1 | rs3917084 | AA | AG | GG | AA | AG | GG |
|  |  | 404 | 22 | 0 | 172 | 6 | 0 |
| Intron 1 | IVS+17C>T | CC | CT | TT | CC | CT | TT |
|  |  | 402 | 30 | 0 | 163 | 15 | 0 |
| Exon 4 | rs2303751 | AA | AG | GG | AA | AG | GG |
|  |  | NA | NA | NA | 49 | 21 | 11 |
| Exon 4 | EX4+89C>T | CC | CT | TT | CC | CT | TT |
|  |  | NA | NA | NA | 87 | 1 | 0 |
| Intron 5 | IVS5-105T>A | TT | TA | AA | TT | TA | AA |
|  |  | 299 | 0 | 0 | 111 | 2 | 0 |
| Exon 6 | EX6+96A>T | AA | AT | TT | AA | AT | TT |
|  |  | 298 | 0 | 0 | 156 | 1 | 0 |
| Exon 6 | EX6+483T>C | TT | TC | CC | TT | TC | CC |
|  |  | 427 | 0 | 0 | 185 | 0 | 1 |
| Exon 6 | rs41268421 | GG | GT | TT | GG | GT | TT |
|  |  | 383 | 34 | 1 | 170 | 9 | 1 |
| Exon 6 | rs1017 | AA | AT | TT | AA | AT | TT |
|  |  | 182 | 192 | 51 | 68 | 82 | 35 |
