# Supplementary material for: Common Variation in ISL1 Confers Genetic Susceptibility for Human Congenital Heart Disease
Source: PLoS One. 2010 May 26;5(5):e10855. doi: 10.1371/journal.pone.0010855 (PMC2877111; doi:10.1371/journal.pone.0010855)
Supplement: Table S3 — Ancestral informative markers available for stage 2 subjects. (0.05 MB DOC) [file pone.0010855.s007.doc]

**Table S3. Ancestral informative markers available for stage 2 subjects**

| **SNP** | **Chromosome** | **Position** |
| --- | --- | --- |
| rs418752 | 5 | 4701002 |
| rs6892396 | 5 | 10386138 |
| rs152363 | 5 | 14809567 |
| rs930072 | 5 | 36701828 |
| rs2910798 | 5 | 37771606 |
| rs277414 | 5 | 41963180 |
| rs7712760 | 5 | 64075218 |
| rs299098 | 5 | 68755507 |
| rs874973 | 5 | 72773651 |
| rs226201 | 5 | 81607602 |
| rs933688 | 5 | 90798504 |
| rs2662258 | 5 | 98153430 |
| rs3317 | 5 | 112240050 |
| rs1382342 | 5 | 115589027 |
| rs3533 | 5 | 126231405 |
| rs2240525 | 5 | 131343783 |
| rs2241583 | 5 | 132589176 |
| rs35294 | 5 | 142339220 |
| rs2400228 | 5 | 146049791 |
| rs165335 | 5 | 150858370 |
| rs246877 | 5 | 156499384 |
| rs4976539 | 5 | 167569852 |
| rs2270895 | 5 | 169400821 |
| rs1363682 | 5 | 174529029 |
| rs632994 | 5 | 175053504 |
| rs2913851 | 5 | 177607823 |
