# Supplementary material for: Common Variation in ISL1 Confers Genetic Susceptibility for Human Congenital Heart Disease
Source: PLoS One. 2010 May 26;5(5):e10855. doi: 10.1371/journal.pone.0010855 (PMC2877111; doi:10.1371/journal.pone.0010855)
Supplement: Table S4 — ISL1 associations with risk of HLHS and TGA in white populations. (0.03 MB DOC) [file pone.0010855.s008.doc]

**Table S4. *ISL1* associations with risk of HLHS and TGA in white populations**

|  | **Stage 1** | | **Stage 2*** | | **Combined†** | |
| --- | --- | --- | --- | --- | --- | --- |
|  | **OR (95% CI)** | **P value** | **OR (95% CI)** | **P value** | **OR (95% CI)** | **P value** |
| rs1017 | 2.04 (1.17 – 3.56) | 0.012 | 1.36 (0.96 – 1.94) | 0.086 | 1.48 (1.08 – 2.04) | 0.016 |
| A-C-T | 2.27 (1.22 – 4.24) | 0.010 | 1.35 (0.90 – 2.04) | 0.15 | 1.62 (1.12 – 2.35) | 0.0099 |

* Analyses controlled for center

† Analyses controlled for center and stag
