# Supplementary material for: Common Variation in ISL1 Confers Genetic Susceptibility for Human Congenital Heart Disease
Source: PLoS One. 2010 May 26;5(5):e10855. doi: 10.1371/journal.pone.0010855 (PMC2877111; doi:10.1371/journal.pone.0010855)
Supplement: Table S5 — CHD diagnoses among whites with ACT haplotype. (0.07 MB DOC) [file pone.0010855.s009.doc]

**Table S5. CHD diagnoses among whites with ACT haplotype.**

|  | **Stage 1** | **Stage 2** | | | **Combined** |
| --- | --- | --- | --- | --- | --- |
|  | **US** | **US** | **Canada** | **Netherlands** |  |
|  | **n=54** | **n=113** | **n=36** | **n=272** | **n=475** |
| **Diagnosis** | **n (%)** | **n (%)** | **n (%)** | **n (%)** | **n (%)** |
| ALCAPA |  | 2 (1.77) |  |  | 2 (0.42) |
| ASD, Primum |  | 4 (3.54) |  |  | 4 (0.84) |
| ASD, Secundum | 1 (1.85) | 8 (7.08) |  |  | 9 (1.89) |
| ASD, Sinus Venosus |  | 4 (3.54) |  | 5 (1.84) | 9 (1.89) |
| AVSD, Complete | 2 (3.70) | 13 (11.50) |  | 14 (5.15) | 29 (6.11) |
| AVSD, Incomplete |  | 2 (1.77) |  | 57 (20.96) | 59 (12.42) |
| Aortic stenosis, Valvular | 3 (5.56) | 8 (7.08) |  |  | 11 (2.32) |
| Atrial abnormality, other | 2 (3.70) |  | 1 (2.78) |  | 3 (0.63) |
| CCTGA (L-TGA) | 2 (3.70) | 1 (0.88) | 1 (2.78) | 24 (8.82) | 28 (5.89) |
| Coarctation of the aorta | 4 (7.41) | 5 (4.42) | 4 (11.11) |  | 13 (2.74) |
| Cor triatriatum |  | 1 (0.88) |  |  | 1 (0.21) |
| D-TGA | 10 (18.52) | 10 (8.85) | 3 (8.33) | 109 (40.07) | 132 (27.79) |
| DORV | 2 (3.70) | 6 (5.31) |  |  | 8 (1.68) |
| Ebsteins anomaly | 1 (1.85) |  |  |  | 1 (0.21) |
| HLHS (AS,AA:MS,MA) | 10 (18.52) | 17 (15.04) | 15 (41.67) |  | 42 (8.84) |
| Mitral stenosis | 1 (1.85) | 1 (0.88) |  |  | 2 (0.42) |
| Pulmonary atresia | 5 (9.26) | 2 (1.77) | 12 (33.33) | 34 (12.50) | 53 (11.16) |
| Pulmonary stenosis |  | 3 (2.65) |  |  | 3 (0.63) |
| Single ventricle (DILV) | 2 (3.70) | 2 (1.77) |  |  | 4 (0.84) |
| TAPVC |  | 2 (1.77) |  | 5 (1.84) | 7 (1.47) |
| TOF | 3 (5.56) | 10 (8.85) |  |  | 13 (2.74) |
| Tricuspid atresia | 1 (1.85) | 1 (0.88) |  | 22 (8.09) | 24 (5.05) |
| Truncus arteriosus | 3 (5.56) | 3 (2.65) |  | 2 (0.74) | 8 (1.68) |
| VSD, Conoventricualr | 2 (3.70) | 8 (7.08) |  |  | 10 (2.11) |
