# Supplementary material for: Common Variation in ISL1 Confers Genetic Susceptibility for Human Congenital Heart Disease
Source: PLoS One. 2010 May 26;5(5):e10855. doi: 10.1371/journal.pone.0010855 (PMC2877111; doi:10.1371/journal.pone.0010855)
Supplement: Table S6 — CHD diagnoses among blacks/African Americans with GCT haplotype. (0.05 MB DOC) [file pone.0010855.s010.doc]

**Table S6. CHD diagnoses among blacks/African Americans with GCT haplotype.**

|  | **Stage 1** | **Stage 2** | **Combined** |
| --- | --- | --- | --- |
|  | **US** | **US** |  |
|  | **n=24** | **n=18** | **n=42** |
| **Diagnosis** | **n (%)** | **n (%)** | **n (%)** |
| ASD, Primum | 2 (8.33) |  | 2 (4.76) |
| ASD, Secundum | 4 (16.67) | 1 (5.56) | 5 (11.90) |
| ASD, Sinus Venosus |  | 1 (5.56) | 1 (2.38) |
| AVSD, Complete |  | 2 (11.11) | 2 (4.76) |
| Aortic stenosis, Valvular |  | 1 (5.56) | 1 (2.38) |
| Coarctation of the aorta | 4 (16.67) | 1 (11.11) | 5 (11.90) |
| DORV |  | 2 (11.11) | 2 (4.76) |
| HLHS (AS,AA:MS,MA) | 4 (16.67) | 1 (5.56) | 5 (11.90) |
| Mitral stenosis | 2 (8.33) |  | 2 (4.76) |
| Pulmonary atresia |  | 2 (11.11) | 2 (4.76) |
| Single ventricle (DILV) | 2 (8.33) |  | 2 (4.76) |
| TOF |  | 1 (5.56) | 1 (2.38) |
| Tricuspid atresia |  | 1 (5.56) | 1 (2.38) |
| Truncus arteriosus |  | 1 (5.56) | 1 (2.38) |
| VSD, Conoventricualr | 4 (16.67) | 4 (22.22) | 8 (19.05) |
| VSD, Muscular | 2 (8.33) |  | 2 (4.76) |
